# Editorial: Functionalization at Nanoscale to Enhance Specific Biological Activities

**DOI:** 10.3389/fbioe.2019.00178

**Published:** 2019-07-25

**Authors:** Piergiorgio Gentile, Emilio I. Alarcon, Mauro Alini

**Affiliations:** ^1^School of Engineering, Newcastle University, Newcastle upon Tyne, United Kingdom; ^2^Department of Biochemistry, Microbiology, and Immunology, University of Ottawa, Ottawa, ON, Canada; ^3^University of Ottawa Heart Institute, Ottawa, ON, Canada; ^4^AO Research Institute Davos, Davos, Switzerland

**Keywords:** nanoscale, surface topography, bio-functionalization, drug delivery, cell response

This Research Topic collects different contributions on nanotechnology-based strategies for controlling and fine-tuning biomaterials' properties at the nanoscale. Such modification allows the production of 3D environments with improved cell compatibility and ultimately functional tissue repair. In this Research Topic, we also present new insights on nanoengineered drug delivery systems.

The first article in this Research Topic (Casanellas et al.), introduces the recent advances in 3D nanoengineered biomaterials for musculoskeletal tissue regeneration, highlighting the different methodologies used to process those. The authors show both conventional and non-conventional technologies to manufacture 3D scaffolds with a fine control of the nanoarchitectural and compositional template for improved tissue integration. Nanofibers in particular had a high surface area, which is optimal for cell adhesion, allowing an interconnected porosity for assisting nutrient transfer and cellular waste. Nanofibrous scaffolds can be produced by conventional electrospinning technology which represents a versatile and widely used method to manufacture nanofibrous scaffolds. Within the non-conventional technologies, 3D printing allows controlling the design and manufacturing of scaffolds with complex structures and intricate geometries that can better mimic the nano- and micro- architecture of the tissues. Furthermore, the addition of nanomaterials during or after the bioprinting process can enhance the scaffold cytocompatibility, tune the physico-chemical and mechanical properties, and direct cellular behavior. Direct nanoscale bioprinting therefore represents a new interesting scenario that better mimics the nanofeatures and nanostructure of the musculoskeletal tissue.

Within the proposed nanoengineered biomaterials, the use of cellulose has recently caught the attention of researchers, due to its tuneable physico-chemical, and mechanical properties, allowing the use of an ideal candidate for scaffold manufacturing. As discussed by Hickey and Pelling, cellulose can be found abundantly in nature and is produced easily. Cellulose-based materials therefore establish a low-cost platform. Moreover, cellulose-based materials satisfy the key criteria for biomedical application: bioactivity, biomechanics and extreme biocompatibility. In their review, the authors provide an up-to-date summary of the field status of cellulose-based nanomaterials in the context of bottom-up strategies of tissue engineering (e.g., skin and wound dressing, bone tissue, blood vessels, and neural applications). Hickey and Peeling then highlight the influence of different nanostructures between bacterial and plant-based cellulose on the biological response of cells. These materials therefore have great potential to become the next standard in biomaterial generation due to their versatility and the diversity of biochemical and physical properties.

Continuing with the application of nanotechnologies, it is extensively accepted that nano-textures applied on construct surfaces can strongly influence some biomaterial characteristics, such as wettability, protein absorption and cellular and/or bacterial adhesion. The importance of the surface functionalization is tackled by Liverani et al. who demonstrates the feasibility of different nano-functionalization strategies to modify the surface of electrospun membranes obtained using non-toxic solvents. Their outcomes show suitable results for all the functionalized polycaprolactone (PCL)-based meshes, with respect to the bare PCL, with better cell behavior on the membranes functionalized with the surface entrapment (after hydrolysis) of fibronectin. This extracellular matrix protein is fundamental for nanoscale ligand-integrin affinity, and is largely used for the functionalization of electrospun meshes as well as for coating cell culture plates in order to enhance cell adhesion and spreading, in products already available on the market.

In another approach, to nano-functionalization with protein and peptides, Al-Azzawi et al. propose the potential improvement of drug uptake through the design and synthesis of low generation lysine dendrons with further functionalization with an ApoE-derived peptide (AEP) ligand. This methodology intends to enhance cellular uptake and drug delivery targeting to the brain in the treatment of neurodegenerative diseases (Al-Azzawi et al). The high density of the terminal functional groups of the dendrimers is a key characteristic, as it allows several attachment sites at the nanoscale for drugs or other biomolecule complexation. In their study, the dendrimers as nanoscale carriers show a strong ability to be internalized by brain endothelia within a receptor-mediated transcytosis (RMT) approach. This type of internalization represents the first step of carrying bioactive molecules into the brain and has an attractive potential for novel therapeutics in the central nervous system.

Finally, a chemical surface treatment for titanium and its alloys is presented in Ferraris et al. who produced and characterized a Ti-oxide layer. Such a surface has a high density of the hydroxyl group, present in the form of a sponge like nanotexture. The treated titanium-based surfaces: (1) present enhanced bioactivity in simulated body fluid solution, encouraging the precipitation of new apatite crystals, and (2) determines an increase in sample anti-adhesive properties; in particular, a substantial inhibition in terms of bacterial viability and metabolism is evidenced for two different bacterial strains, *S. Aureus* and *A. Actinomycetemcomitans*, without compromising the material cytocompatibility.

We hope that readers find this Research Topic to be a useful collection of articles in the emerging field of nanoscale functionalization and the manufacturing of medical systems in tissue engineering and drug delivery applications.

## Author Contributions

All authors listed have made a substantial, direct and intellectual contribution to the work, and approved it for publication.

## Conflict of Interest Statement

The authors declare that the research was conducted in the absence of any commercial or financial relationships that could be construed as a potential conflict of interest.

